# Exploring women's experiences and needs during the climacteric to enhance positive mental health and self-care

**DOI:** 10.3389/fgwh.2026.1733517

**Published:** 2026-07-22

**Authors:** Maria Aurelia Sánchez-Ortega, Sara Sanchez-Balcells, Marta Prats-Arimon, Carmen Moreno-Arroyo, Núria Rodríguez-Ávila, Elena Maestre-Gónzalez, Esther Crespo-Mirasol, Zaida Agüera, Encarna López, Cristina Martínez-Bueno, Teresa Lluch-Canut, Montserrat Puig-Llobet

**Affiliations:** 1 Department of Fundamental Professionals and Health Management, Health Science and Well-being Faculty, University of Vic, Vic, Spain; 2 Department of Public Health, Mental Health and Maternal-Child Nursing, Faculty of Nursing, University of Barcelona, Health Sciences Campus Bellvitge, L’Hospitalet de Llobregat, (Barcelona), Spain; 3 Department of Clinical and Fundamental Nursing, Faculty of Nursing, University of Barcelona, Health Sciences Campus Bellvitge, Hospitalet de Llobregat (Barcelona), Spain; 4 NURSEARCH Research Group, Emerging Research Group 2021 SGR 1083: Nursing Care in Mental Health, Psychosocial and Complexity, University of Barcelona, Hospitalet de Llobregat, Spain; 5 Faculty of Sociology, University of Barcelona, Barcelona, Spain; 6 Sexual and Reproductive Healthcare (ASSIR), Catalan Institute of Health, Barcelona, Spain; 7 Faculty of Nursing, GRASSIR Research Group 2021-SGR-793, Research Group on Sexual and Reproductive Healthcare, University of Barcelona, Hospitalet de LLobregat, Spain; 8 Faculty of Nursing, University of Barcelona, Hospitalet de Llobregat, Spain; 9 GIRISAME Research Group, International Group of Mental Health Nursing Researchers, Madrid, Spain; 10 Nursing Interventions in Complex Chronic Patient Nursing, Faculty of Nursing, University of Barcelona, Hospitalet de Llobregat, Spain

**Keywords:** aging symptoms, climacteric, positive mental health, self-care, women

## Abstract

**Background:**

The climacteric is a stage in women's lives that, even today, remains taboo in many regions of the world where social contexts are otherwise highly advanced. Although women's experiences have generated growing interest there is still a need for greater understanding. Learning about and studying the experiences and needs that determine women's emotional well-being during the various stages of the climacteric provides a knowledge base that allows future research to design evidence-based, high-quality health promotion and intervention programs aimed, from a holistic perspective, at fostering well-being during this stage of life. In this sense, a project was proposed to explore these experiences and needs and to co-create a Decalogue for Positive Mental Health during the climacteric.

**Methods:**

A qualitative, interpretive phenomenological study was conducted. Data were collected through an online questionnaire and two focus groups (*n* = 12) between February and April 2025. The sample was recruited by convenience. Inclusion criteria were women currently experiencing the climacteric who were available to participate in the focus groups. All discussions were recorded, transcribed verbatim, and analyzed using ATLAS.ti version 25 through content analysis.

**Results:**

Three main categories emerged: symptoms, self-care, and aesthetic pressure. A total of 59 codes were identified, and sentiment analysis was conducted, showing predominantly neutral and negative results across the three categories.

**Conclusions:**

The modern lifestyle has transformed the profile of women experiencing the climacteric. Today, they are more aware of this stage and demand clear information and standardized protocols to better understand it. Furthermore, self-care and positive mental health have consolidated as key elements to navigate this transition period with well-being.

## 1 Introduction

Gender bias in health research is evident. Morbidity differs according to gender and sex, with both physiological and psychosocial variations. Despite advances in integrating the gender perspective into health sciences, an androcentric focus continues to dominate the research priorities. Consequently, it remains necessary to study and deepen understanding of the changes that occur in women's health.

The climacteric is the transitional period in a woman's life from the reproductive to the non-reproductive stage. It typically occurs between the ages of 42 and 58, either physiologically or because of medical treatments (1). This life phase involves numerous physical, sexual, and psychological changes that can affect quality of life (2, 30). The process begins with progressive irregularity in menstrual cycles, leading eventually to permanent cessation of menstruation (3), with women being considered postmenopausal after 12 consecutive months without menstruation. Currently, due to increased life expectancy, it is estimated that women spend approximately 40% of their lifespan in the postmenopausal stage, which in some cases may account for up to 30 years of life (4, 29).

A wide variety of symptoms occur during this stage. Each woman experiences her transition to menopause differently, and this transition involves a wide range of physiological, psychological, and sociocultural symptoms. It is also important to emphasize the duration of these symptoms, as in some cases they may persist for long periods and become severe (37–39, 43). Physically, the most frequent manifestations are vasomotor symptoms such as hot flashes, night sweats, and sudden waves of heat; musculoskeletal symptoms such as joint pain due to reduced estrogen levels; sleep disturbances with nocturnal awakenings; genitourinary symptoms such as vaginal dryness and dyspareunia; memory difficulties or brain fog (5, 6); weight gain (7, 8); as well as palpitations and urinary, vaginal, and sexual problems. Psychologically, studies indicate that during the transition to menopause, women may experience lower self-esteem and greater vulnerability to depression and anxiety (9). Self-care deficits also arise due to lack of knowledge about symptoms, care, and available resources during the climacteric period (10, 28, 33).

Depending on the sociocultural context, menopause is experienced differently (26). In wealthier countries, it is understood as a negative process that must be medicalized through hormone replacement therapy in order to prevent aging, a life stage with which it is commonly associated (41). In contrast, in poorer countries it is understood as a natural aging process and is accepted with positive aspects, such as the cessation of menstruation (11, 27, 35, 40, 42). Social perceptions of the climacteric have also shaped women's experiences. Historically, this stage has been viewed as an infertile and declining period, associated with aging and negative connotations, often treated as taboo and avoided even by women themselves. Additionally, women face aesthetic pressure, which continues during the climacteric, compounding the stigma of aging. Such aesthetic pressure impacts self-esteem and can trigger both physical and mental health problems (12). The Plan of Action to Combat Aesthetic Pressure 2023–2026, developed by the Generalitat de Catalunya, emphasizes the need to destigmatize menopause and raise social awareness.

The lack of media representation further contributes to the discrimination faced by women during this life stage (13). According to the World Health Organization (WHO), between 60% and 70% of women in the climacteric stage lack basic knowledge about its physical and psychological impact. Among the many physical changes, weight gain and increased risk of obesity are common, which may lead to adverse health effects. Combined with aesthetic pressure and ageism, these factors can also result in eating disorders during this period (14, 34).

Another important aspect is the caregiving role that many women assume during this stage of life. The current demographic situation increases the need for care for dependent older adults, and it is mostly women—often family members—who provide such care. As informal caregivers, this generates social, economic, and physical burdens. Furthermore, the sustained stress of caregiving can have serious consequences on physical and mental health (15, 25).

The conceptual framework of this study centers on self-care and positive mental health (PMH), understanding their interrelationship and the perspective surrounding the climacteric stage within our social context. Self-care is framed within Dorothea Orem's General Theory of Self-Care Deficit, which defines self-care as “the deliberate actions individuals take to maintain life, health, and well-being, and to consistently respond to their health needs.” PMH follows the model of Maria Teresa Lluch, who defined it as a multifactorial construct comprising six interrelated factors: (1) Personal satisfaction, (2) Prosocial attitude, (3) Self-control, (4) Autonomy, (5) Problem-solving and self-actualization, and (6) Interpersonal relationship skills. Lluch (16, 32) proposed a Decalogue of Positive Mental Health that summarizes these factors in ten inspirational statements promoting a positive approach to life. Lluch's approaches reinforce the shared idea that one must live and experience each moment by acting appropriately in each case, allowing the expression of feelings, and channeling and managing them coherently.

Empirical evidence supports a bidirectional relationship between self-care and PMH: greater self-care leads to higher PMH and vice versa (36). Intervention programs such as PIPsE, designed to enhance PMH in people with chronic illnesses (17), have shown promising results. Based on this framework, this study aimed to explore women's experiences and needs during the climacteric to enhance positive mental health and self-care, and to co-create a Decalogue for Positive Mental Health during the Climacteric.

## 2 Materials and methods

A qualitative study with an interpretive phenomenological approach was conducted, following the Standards for Reporting Qualitative Research (SRQR) checklist. One objective was to explore women's experiences and needs during the climacteric—a life stage historically given little attention due to age and gender biases and still often treated as taboo. Another objective was to co-create a Decalogue based on positive mental health aimed at people in the climacteric stage. The study was approved by the Research Ethics Committee of the University of Barcelona (Institutional Review Board IRB00003099: CER032510).

### 2.1 Participants and setting

A total of 12 participants (*n* = 12) were selected using convenience sampling. Inclusion criteria were women in the climacteric who agreed to participate and had availability to attend the focus groups. The only exclusion criterion was language barrier. Participants received full information about the study in person, and informed consent was obtained. Only one person declined to participate due to the inability to attend the focus groups.

### 2.2 Data collection

Data were collected using three tools: (1) individual online interviews via a sociodemographic questionnaire; (2) a self-administered sheet to co-create a Decalogue for Positive Mental Health during the climacteric based on Lluch (16); and (3) two focus groups recorded with prior consent. Data were collected between February and April 2025. Two meetings were held with the focus groups using the planned discussion method.

Focus Group 1 covered: (a) symptoms experienced and associated limitations; (b) perceived self-care needs, both physical and emotional; and (c) perceived aesthetic pressure and impact on self-esteem.

Focus Group 2 used data from the first session and the co-creation grid to discuss and reach consensus on the Decalogue statements.

Both focus groups were led by two doctoral-level university researchers with qualitative research expertise, lasting 120 min, and were conducted online. Data collection concluded upon reaching theoretical saturation. The sequence was: individual interviews; first focus group; co-creation grid; second focus group.

The sequence of data collection was as follows: first, the individual interviews and the content of the original Positive Mental Health Decalogue were sent; second, the first focus group was conducted; third, the template used to collect the co-creation data for the positive mental health decalogue was sent; and fourth, the second focus group was conducted. During this second meeting, and in relation to the creation of the climacteric decalogue based on positive mental health, a database was created compiling the participants' contributions. A consensus had to be reached on ten statements that would compose the decalogue, based on the initial Positive Mental Health Decalogue created by Dr Lluch in 2011. Initially, the content was agreed upon through a list containing different options, and the participants subsequently revised this first list until a consensus was reached on a single option for each statement.

### 2.3 Data analysis

Data were analyzed using an interpretive phenomenological approach (18) with ATLAS.ti 25. A deductive-inductive framework was applied. Researchers transcribed the recordings verbatim and conducted iterative readings to identify emerging themes. Rigor followed Lincoln and Guba's criteria. Participants later reviewed their transcripts to make corrections if necessary. Coding was performed independently by the two researchers who conducted the groups.

Coding was carried out using the free-coding function, and segments were generated within the paragraphs to establish the codes. These were organized by colors in order to identify the main categories and were subsequently reviewed until saturation was reached, arranging overlapping codes and reducing them to a final total of 59 codes after the merging of redundant codes. Sentiment analysis was also performed, classifying sentiments as positive, neutral, and negative.

For the Decalogue creation, participants' contributions were compiled into a database. Consensus was reached on ten statements, starting from Lluch's original 2011 Decalogue and refining options to a single agreed-upon formulation per statement.

## 3 Results

### 3.1 Participant characteristics

The sample comprised twelve women aged 48–58 years, all residents of the province of Barcelona (rural and urban), all currently employed. This results can be observe in Table 1.

**Table 1:** Participant profile.

Participants	Age	Profession
P1	58	Sociologist
P2	50	Chemist
P3	52	Shop assistant
P4	55	Administrative staff member
P5	52	Shop assistant
P6	53	Nutritionist
P7	48	Teacher
P8	58	Midwife
P9	52	Administrative staff member in accounting
P10	51	Nurse
P11	56	Social worker
P12	49	Audiovisual graduate
*n* = 12	52.8	

Authors' own elaboration.

### 3.2 Main categories and codes

Three main thematic categories emerged: (1) Symptoms, (2) Self-care, and (3) Aesthetic pressure. In total, 59 codes were identified: 14 for Symptoms, 11 for Self-care, and 34 for Aesthetic pressure.

#### 3.2.1 Category 1: symptoms

Findings in this category relate to the symptoms experienced during the climacteric stage. These are numerous and vary in intensity among individuals. Both physical and emotional manifestations emerged from the data.

With respect to physical symptoms, participants reported hot flashes, insomnia, weight gain, loss of muscle mass, and physical limitations associated with general discomfort. At the emotional level, participants described body shame, mood swings, apathy, mental fog, and feelings of invisibility. They also emphasized that the physical changes occurring in women during this stage do not occur in men, leading to differences in the way aging is experienced.

Physical aspects:

From the age of 48, it's been relentless; it all hit me at once. (P1)

Hot flashes, insomnia—spending all night tossing and turning because they wouldn't let me sleep. (P3)

We're getting older and losing muscle mass; menopause is the turning point. (P11)

I've gained a lot of weight and feel more limited because of the discomfort. (P2)

Men get more interesting with age; we become invisible. (P4)

Emotional symptoms also emerged, as menopause was associated with the conclusion of a reproductive stage traditionally linked to youth and female fulfillment.

Sexually, I was separating and dating again; the desire was still there, but getting naked in front of someone new was very difficult. (P5)

You feel very alone in this situation. (P4)

I have constant mood swings. (P8)

I don't feel like going out like I used to. (P10)

I suddenly became invisible—I didn't even notice the moment it happened. (P1)

#### 3.2.2 Category 2: self-care

Findings in this category revealed a focus on preventive self-care. Participants highlighted the importance of having prior information to enable timely responses and to plan a more individualized climacteric process. Discussion also addressed the caregiving role that women assume at the social level: they are often caregivers for children, partners, and older adults, yet in most cases, self-care is neglected. Women tend to care for everyone except themselves.

Before it starts, we should have a care plan to organize ourselves. (P9)

You can't eat like before—you gain weight and feel bloated. (P2)

They give you a gel, or you go to the pharmacy for sleeping pills. (P12)

It would be better to have a guide to know what to do because it's different for each of us. (P12)

It would be ideal if someone explained what can happen, gave us tools and resources. (P3)

We need a preventive guide; I wasn't paying attention to the signs because I was sandwiched (referring to caring simultaneously for parents and children) and didn't listen to my body. I was going full speed for everyone except myself. (P6)

We're a new profile—more educated and more demanding; we want answers. If I'm 48 and will live to 80, how long will I be in menopause? (P7)

A guide could remove guilt—it's never too late. Keep a positive mindset that you are always in time to do something about it. (P2)

In the end, it's all learning—no need to blame ourselves. (P12)

We're in a sandwich situation — our children pull us one way and our parents the other, and we have no time to take care of ourselves. (P6)

I'd like to share a thought — among mammals, only whales and female elephants experience menopause. It is believed that once they are no longer reproductive, these females take on caregiving roles and transmit their knowledge to the younger members of the group. They are considered the most important, precisely because of the wisdom and experience they have accumulated. (P1)

You keep holding on and holding on, and you don't know where the limit is. (P7)

There are demands coming from all sides; sometimes I just want to shout: enough! I need an hour for myself. (P9)

Families are changing—some women have their first child at 48, so by 50 they face a new context. (P10)

#### 3.2.3 Category 3: aesthetic pressure

At some point, you just become invisible. (P8)

Do you feel aesthetic pressure in society? How do you experience it? (P4)

I think I look fine. (P2)

I feel I should take better care of myself. (P8)

I don't care what people say. (P9)

I'm losing muscle mass. (P3)

There are things I don't like, but I try to see the positive side. (P10)

It affects me because I feel heavy—it crushes my self-image. (P12)

I didn't take good care of myself when I should have, especially with food, and now I'm afraid. (P6)

The pressure comes from ourselves; we need an inner process of self-acceptance. (P5)

It's more internal than external. (P4)

After my separation I felt fine, but later I realized I wasn't doing things the same as before. (P11)

We should be warned to take care when the first symptoms appear—control your diet and do exercise. (P7)

There's social interest in menopause now; in motherhood people help you, but with this you feel very alone. (P7)

Social support is needed— women in this stage are often perceived as irritable, and they go through it feeling very much alone. (P5)

Socially, it's not talked about; you only hear about it from your mother. (P1)

After the pandemic, at work we decided to discuss a topic related to women. Someone suggested talking about the menstrual cup, but the more experienced women said no — that we should talk about menopause. (P10)

Youth is the standard. (P4)

You look in the mirror, and your body isn't the same. (P2)

There's no point of return—only a point of acceptance. (P8)

I decided not to dye my hair—that's a social choice. (P6)

It's a life cycle, but people don't reflect on it. (P12)

I knew it was coming, but somehow, I didn't think it would happen to me — I thought I'd be spared, but I wasn't. (P11)

I was aware of physical changes, but not cognitive ones. (P8)

You think you're fine when it hasn't started yet, but when it finally comes — even though you know about it — that's when you truly experience it. (P2)

There are differences between men and women at this stage. (P5)

The environment affects you. (P2)

I didn't talk about this before, but we've moved forward and now we share more. (P9)

Talking with others helps—you can share stories. (P10)

I reclaimed menopause. (P3)

Some friends don't want to have any more periods. (P7)

You think that when menstruation ends, it's the end of an era, but that's not the case. (P11)

Table 2 presents the codes identified for each of the main categories.

**Table 2:** Principal categories and codes.

Category	Codes	
Category 1: Symptoms	1. Emotional adaptation 2. Emotional support 3. Social well-being 4. Emotional change 5. Gender differences 6. Lack of motivation 7. Frustration	8. Gender and menstruation 9. Sexual identity 10. Physical discomfort 11. Health problems 12. Physical health 13. Physical symptoms 14. Loneliness
Category 2: Self-care	1. Self-esteem 2. Well-being 3. Social change 4. Care 5. Guilt 6. Gender stereotypes	7. Resource management 8. Innovation 9. Personalization 10. Prevention 11. Health
Category 3: Aesthetic pressure	1. Acceptance 2. Adaptation to situations 3. Social support 4. Self-acceptance 5. Self-confidence 6. Self-care 7. Self-evaluation 8. Self-image 9. Emotional change 10. Communication 11. Awareness 12. Grief over loss 13. Expectations 14. Shared experiences 15. Gender experiences 16. Lack of communication 17. Uncertainty 18. Misconceptions about menstruation	19. Indifference 20. Social interest 21. Youth 22. Motherhood 23. Fear 24. Social norms 25. Perception of menstruation 26. Self-perception 27. Social perception 28. Positive perception 29. Health concerns 30. Reflection 31. Advocacy 32. Health and well-being 33. Taboos 34. Communicative transformation

Authors' own elaboration. Code matrix structured from the qualitative content analysis conducted using ATLAS.ti 25.

Regarding sentiment analysis, as can be observed in Table 3, the results by category were as follows. Category 1: Symptoms was the category that gathered the highest number of neutral comments (85%), with 15% negative comments and no positive comments reported. The women stated that the symptoms experienced during menopause were neither positive nor pleasant, although they maintained an adaptive attitude in which, despite not generating enthusiasm, the situation was accepted. Category 2: Self-care showed the same proportion of comments as Category 1 for the interviewees, with both accounting for 27.4% of comments; however, it presented a higher level of negative sentiment than the previous category (25%). Category 3: Aesthetic pressure was the category with the greatest weight (45.2%), representing women's feelings regarding the physical changes experienced during this stage, and it also showed the highest rate of negative sentiment (33%).

**Table 3:** Results of sentiment analysis by category.

Categories	Positive/%	Neutral/%	Negative/%	Total quotes	%
Category 1: Symptoms	0 (0%)	17 (85%)	3 (15%)	20	27.4
Category 2: Self-care	0 (0%)	15 (75%)	5 (25%)	20	27.4
Category 3: Aesthetic pressure	1 (3%)	21 (64%)	11 (33%)	33	45.2
Total	1 (1.4%)	53 (72.6%)	19 (26%)	73	100

Authors' own elaboration, data obtained through sentiment analysis using ATLAS.ti 25.

### 3.3 Co-creation of the decalogue for positive mental health during the climacteric

Regarding the Positive Mental Health Decalogue in the climacteric stage, Table 4 presents both the original Decalogue and the climacteric-stage Decalogue in parallel form in order to facilitate comparison and understanding.

**Table 4:** Contents of the original positive mental health (PMH) decalogue and the decalogue adapted for the climacteric stage.

Original positive mental health decalogue (16)	Decalogue of positive mental health during the climacteric stage (Co-creation, 2025)
**Recommendation No. 1:** Positively value the good things we have in our lives.	**Recommendation No. 1:** Value the experience accumulated over the years and appreciate this new stage, which may bring greater freedom through the absence of menstruation.
**Recommendation No. 2:** Approach everyday activities with care and affection. Happiness is hidden among us in daily life. Everyday activities (taking the metro, shopping, working, etc.) should not be faced as a punishment or with indifference. We must seek the positive aspects of these activities and approach them with a favorable state of mind.	**Recommendation No. 2:** Dedicate more time to ourselves, manage it better, and stop when necessary in order to regain strength.
**Recommendation No. 3:** Do not be too harsh on ourselves or on others. Tolerance, understanding, and flexibility are beneficial for mental health.	**Recommendation No. 3:** Take care of ourselves, understand ourselves, treat ourselves kindly, avoid placing excessive physical and mental demands on ourselves, and put adverse situations into perspective.
**Recommendation No. 4:** Do not allow negative emotions to block our lives: it is important to become angry when necessary, but not to become overwhelmed by it.	**Recommendation No. 4:** Identify negative emotions in order to confront them, express them verbally or write them in a journal, and learn to channel them through personalized resources such as singing, listening to music, or exercising.
**Recommendation No. 5:** Become aware of the good moments that occur in our lives while they are happening. If our lives contain good moments worth remembering, they must also have been good moments when we experienced them. Therefore, we should enjoy the positive aspects of the present, in addition to remembering the good things from the past and looking forward to good things in the future.	**Recommendation No. 5:** Be aware of what is gained during this stage, enjoy everyday experiences, look back without longing, and embrace this new phase with hope and enthusiasm.
**Recommendation No. 6:** Do not be afraid to cry and feel emotions. It is important to understand the normality of many feelings: if we have experienced disappointment, it is normal to feel discouraged; if we have lost a loved one, it is normal—and mentally healthy—to feel sadness. However, if emotional states become very intense, persistent, or disabling, professional help should be sought.	**Recommendation No. 6:** Allow ourselves to express our feelings to ourselves and to trusted people, and seek supportive spaces for doing so if needed. Recognize the normality of this process due to hormonal changes and consult a professional if necessary.
**Recommendation No. 7:** Seek spaces and activities that help us relax mentally. Each person has their own preferences, resources, and strategies (walking, reading, gardening, doing nothing, talking with friends, etc.).	**Recommendation No. 7:** Identify activities, physical spaces, and people that help us relax, and devote time to enjoying them.
**Recommendation No. 8:** Try to resolve the problems that arise in our lives. If problems accumulate, mental health suffers. Not all problems have ideal solutions, but we should always try to do something to alleviate them. This reflects an active predisposition towards seeking solutions.	**Recommendation No. 8:** Have clear and useful information about this stage in order to understand the changes we are experiencing and those that may come, and to know how to prioritize and cope with them.
**Recommendation No. 9:** Take care of our interpersonal relationships. Let us talk with our loved ones, visit our friends, and share conversations with colleagues, neighbours, and others.	**Recommendation No. 9:** Take care of our interpersonal relationships, value sisterhood, solidarity among women, and women's social networks. Nurture all areas of life by engaging in activities with friends or partners and, when applicable, embracing a new sense of sensuality.
**Recommendation No. 10:** WE MUST NOT FORGET TO COLOR LIFE WITH HUMOR SO THAT IT HAS MORE COLOR.	**Recommendation No. 10:** Introduce humor and look for the amusing side of the new situations that arise.
	Thank you to all women for existing!!!

Authors' own elaboration based on the contents of Maria Teresa Lluch’s Decalogue of Positive Mental Health (2011). Retrieved from: https://chrome-extension://efaidnbmnnnibpcajpcglclefindmkaj/https://diposit.ub.edu/server/api/core/bitstreams/60a68036-4d15-4145-ae4a-738280c5e5ee/content.

## 4 Discussion

The participants' experiences were analyzed within three thematic areas: symptoms and their limitations; self-care needs (physical and emotional); and aesthetic pressure and its effect on self-esteem. This structure resembles that of a Saudi study (19), where similar symptoms and preferences for self-care activities were reported. However, aesthetic pressure appeared less central in our sample compared to the Saudi context in which women felt concerned about a perceived loss of attractiveness, likely reflecting cultural differences.

Comparable findings emerged in a South Asian study (20) in which the results align with those reported in Saudi Arabia regarding aesthetic pressure, uncertainty about bodily changes, and the process of accepting aging. The uncertainty associated with bodily changes and the lived experience of accepting the transition to aging are central themes. This study underscores the role of social constructs, given that the sample comprised Canadian women residing in South Asia. The resulting cultural contrast revealed the need to examine these social dimensions in greater depth.

In a study conducted in China, it was observed that women were reluctant to use hormone replacement therapy, and it was concluded that a comprehensive approach—one that considers both physiological and psychological well-being—would be effective for addressing the menopausal transition (21). In this regard, the findings align with the psychological well-being needs and the desire for knowledge about symptoms reflected in our study, as well as with another study conducted with a sample of African-descendant women, in which the results showed an interest in receiving information about menopause and improving lifestyle (22).

Another study carried out in Greece produced relevant data concerning social factors and their influence on women's perceptions of the intensity of menopausal symptoms. In our study, social factors were linked to self-care and service improvement, as well as to aesthetic pressure, social expectations, perception, and social interaction (31).

The notion of youth as the definition of beauty and the belief that menopause is an unattractive stage of life are consistent with the findings of a study conducted in South Africa (23). Interestingly, the same study also coincided with the view that reaching menopause marks the beginning of a normal and calmer phase than before, thus adding a positive dimension to the physical transition.

Another highly interesting study conducted in Spain (24) proposes ten measures for the public management of the climacteric process, based on governmental actions related to social awareness and support initiatives, educational proposals, and political and regulatory measures. In this regard, it differs from our study: although both works are structured around ten proposals, our *Decalogue* consists of ten inspirational statements grounded in positive mental health, whereas Selva et al.'s work focuses on governmental proposals, and ours on personal ones.

## 5 Limitations

One of the study's limitations relates to the sample, which includes participants only from a specific area of Catalonia and from a predominantly local profile. Although we consider this a good starting point for future research, it would be valuable to include participants from other cultural backgrounds in subsequent discussion groups to broaden perspectives and enrich the findings.

The findings of this study cannot be extrapolated to other countries or communities. However, although they may not be generalizable, they may still meet the conditions for transferability.

## 6 Conclusions

The main conclusions of this study are as follows:

- The climacteric is a unique experience for each woman, though symptoms are often similar.
- Symptoms encompass both physical and emotional domains and are often experienced as personal decline.
- Gender differences are evident: women live the climacteric and its social consequences, whereas men do not; women may feel “invisible” while men are perceived as more “interesting.”
- Aesthetic pressure significantly affects women during this stage, as youth remains the socially desired standard.
- A new profile of women has emerged—more active, with longer life expectancy, and spending more years in this phase.
- At present, women seek greater attention and value the sense of sisterhood among themselves.
- Clear, standardized protocols are needed to provide preventive information for better understanding of the process.
- Self-care is fundamental; preventing self-neglect helps maintain self-esteem and well-being.
- Viewing this stage through the lens of positive mental health frames it as a natural and empowering life phase.
- Ten recommendations were co-created based on and inspired by the original Positive Mental Health Decalogue, forming the Positive Mental Health Decalogue for the climacteric stage.

## 7 Contribution of the article

This article contributes to the scientific community by providing a description of women's experiences during the climacteric stage, an exploration of their emotional state, and a proposal to promote their well-being. It offers two main contributions: first, the identification of the need to develop a preventive guide to help women plan for the onset of this stage; and second, the creation of a *Decalogue for Positive Mental Health* and the recommendations it provides.

## Data Availability

The datasets generated and/or analyzed during the current study are available from the corresponding author upon reasonable request.
